# Pre-discharge neutrophil:lymphocyte ratio and lymphopenia are associated with post-hospital long-term outcomes among survivors of critical illness

**DOI:** 10.1186/s40635-026-00918-0

**Published:** 2026-06-15

**Authors:** Lucas L. Marinho, John C. Marshall, Danielle Jeong, Dennis T. Ko, Patrick R. Lawler

**Affiliations:** 1 McGill University Health Centre, McGill University, 1001 Boulevard Décarie, Montreal, H4A3J1 Canada; 2 University of Toronto, Toronto, Canada; 3 Unity Health, Toronto, Canada; 4 Sunnybrook Research Instittue, Toronto, Canada

**Keywords:** Critical iIlness, Inflammation, Neutrophil, Lymphocyte, Biomarkers

To the Editor,

Critical illness survivors remain at high risk for adverse outcomes after hospital discharge [1]. Risk stratification at discharge remains a gap in post-hospital care. Biomarkers reflecting ongoing immune dysregulation may help identify patients at increased mortality risk after discharge.

Dysregulation of myeloid and lymphoid compartments is a hallmark of diverse critical illness syndromes [2]. Persistent immune dysregulation is associated with higher 1-year mortality after sepsis [3]. Accordingly, among ICU survivors, we evaluated the association between pre-discharge lymphopenia and 1-year mortality using two clinically available biomarkers: the neutrophil:lymphocyte ratio (NLR) and absolute lymphopenia defined by standard thresholds.

Critically ill adults receiving ICU care who survived through hospital discharge (2008–2022) were identified in the MIMIC-IV database [4]—a publicly available single-center database in Boston, US, accessed through granted access and local ethics approval. Exclusions were elective admission, ICU stay < 48 h, discharge to another facility, hematologic malignancy, rheumatic diseases, HIV, and extreme (top/bottom 1%) values.

Exposures were the last biomarker values ≤ 72 h before hospital discharge: (a) the NLR (absolute neutrophil count divided by absolute lymphocyte count [ALC]), and (b) lymphopenia (ALC < 1000 cells/mm^3^). The outcome was 1-year post-discharge all-cause mortality [4], evaluated using adjusted restricted cubic splines (covariates in Fig. 1).

**Fig. 1:**
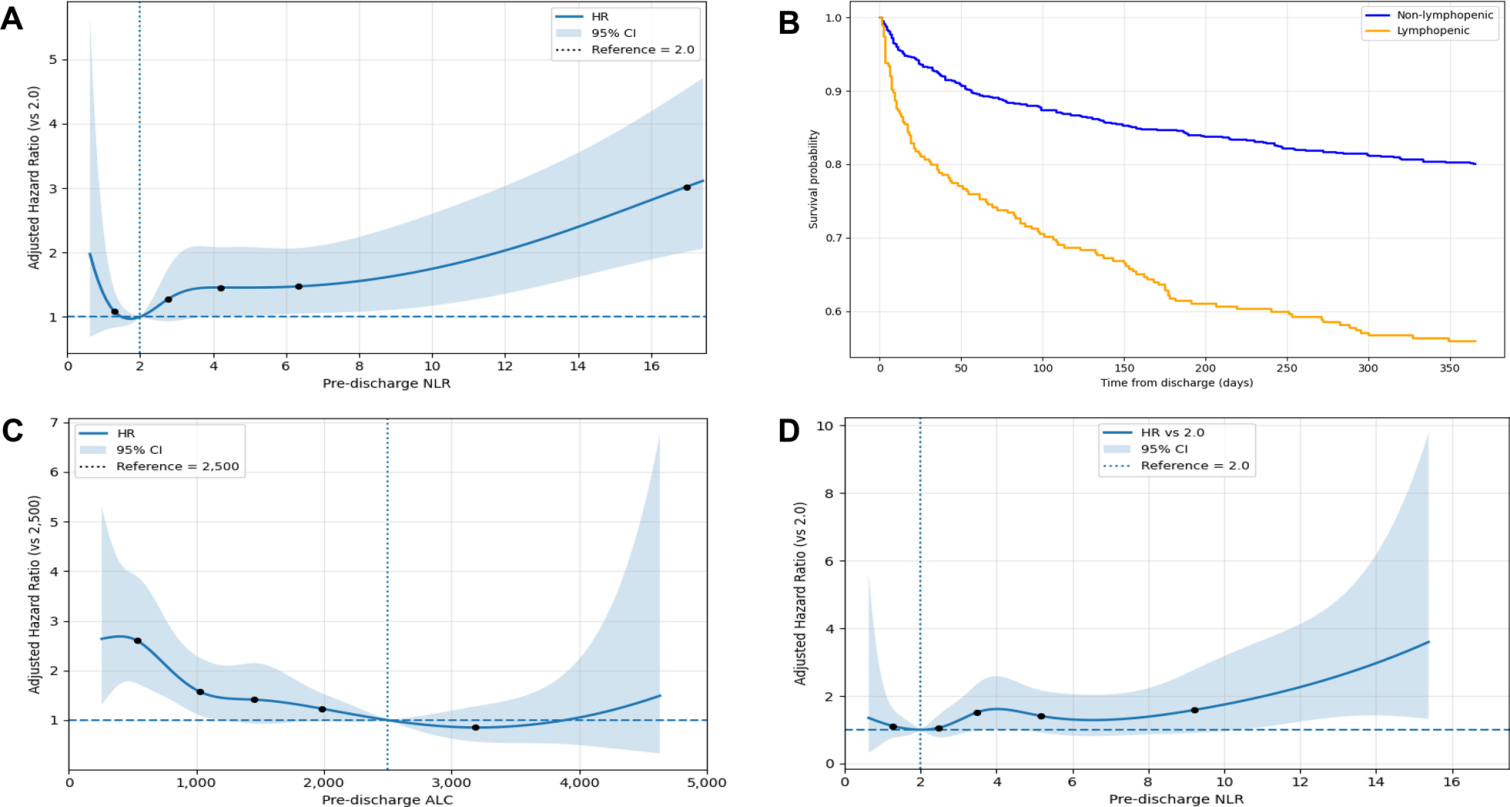
** A** Adjusted* relative hazards for time to death through 365 days by pre-hospital discharge NLR value** (*n* = 1052). **B** Survival after critical illness discharge according to pre-hospital discharge lymphopenia*** (*n* = 1052). **C** Adjusted* relative hazards for time to death through 365 days by pre-hospital discharge ALC value**** (*n* = 1052). **D** Adjusted* relative hazards for time to death through 365 days by pre-hospital discharge NLR value** excluding patients with pre-hospital discharge lymphopenia*** (*n* = 777). *Adjusted for age, sex, baseline weight, Charlson Comorbidity Index, worst SOFA score within 24 hours of ICU admission, ICU length of stay, and need for renal replacement therapy during hospitalization. **Restrictive cubic spline with 5 knots; although there is no established normal range or NLR, a median value of 2.0 has previously been observed in healthy individuals; therefore, this was used as the reference value herein. ***ALC < 1000 cells/mm^3^ ****Restrictive cubic spline with 5 knots; within the normal ALC range (1000–4000 cells/mm^2^), a value of 2500 cells/mm³ was used as the reference. In Panels **A**,** C**, and **D**, circles indicate model knots; In Panel **A**, the figure is truncated at an NLR of 17.5 (95.2th percentile)

Among 20,906 eligible survivors, 1052 had available NLR and ALC. Missingness reasons were not available and may reflect lack of measurement or incomplete ascertainment; therefore, selection bias cannot be excluded. Median (IQR) NLR was 4.2 (2.6–6.8). Lymphopenia was present in 26%. Higher NLR and lymphopenia tracked greater illness severity and comorbidity (Table 1), including malignancy.

**Table 1:** Baseline characteristics^*^ according to pre-discharge NLR tertiles and lymphopenia

	Overall	NLR			Lymphocytes	
		NLR T1	NLR T2	NLR T3	ALC < 1000	ALC ≥ 1000
		(≤ 3.06)	(3.07–5.67)	(≥ 45.28)		
	(*n* = 1052)	(*n* = 351)	(*n* = 351)	(*n* = 350)	(*n* = 275)	(*n* = 777)
Last NLR < 72 h pre-discharge						
NLR	4.20 (2.56–6.81)	2.07 (1.56–2.56)	4.20 (3.49–4.86)	8.55 (6.83–12.91)	7.78 (4.32–12.81)	3.55 (2.34–5.37)
Time from hospital admission to NLR pre-discharge (days)	10 (6–17)	10 (6–18)	10 (6–18)	10 (6–17)	10 (6–17)	10 (6–18)
Time from NLR pre-discharge to hospital discharge (h)	12 (9–29)	12 (9–30)	12 (9–29)	12 (9–26)	12 (9–25)	12 (9–30)
Demographics						
Age (years)	65 (52–76)	60 (44–72)	65 (52–77)	69 (58–77)	69 (59–76)	63 (50–75)
Sex (male)	575 (54.7%)	183 (52.1%)	194 (55.3%)	198 (56.6%)	150 (54.5%)	425 (54.7%)
BMI (kg/m^2^)	27 (24–33)	29 (25–34)	28 (24–33)	26 (23–30)	26 (23–30)	28 (24–33)
Comorbidities						
Charlson comorbidity index	5 (2–7)	4 (2–6)	4 (2–7)	6 (4–8)	6 (4–9)	4 (2–6)
Critical illness						
Sofa score 24 h	4 (2–7)	4 (2–6)	5 (2–7)	4 (2–7)	5 (2–7)	4 (2–7)
ICU length of stay (days)	4 (3–7)	4 (3–7)	4 (3–8)	4 (3–7)	4 (3–6)	4 (3–7)
Hospital length of stay (days)	11 (7–18)	11 (7–18)	11 (7–18)	11 (7–18)	10 (7–18)	11 (7–18)
Vasopressor (first 48 h)	315 (29.9%)	91 (25.9%)	125 (35.6%)	99 (28.3%)	75 (27.3%)	240 (30.9%)
Invasive ventilation	502 (47.7%)	154 (43.9%)	192 (54.7%)	156 (44.6%)	120 (43.6%)	382 (49.2%)
Sepsis	504 (47.9%)	148 (42.2%)	179 (51.0%)	177 (50.6%)	132 (48.0%)	372 (47.9%)
Surgery during hospitalization	424 (40.3%)	108 (30.8%)	155 (44.2%)	161 (46.0%)	118 (42.9%)	306 (39.4%)
Baseline laboratories^**^						
NLR	8.48 (4.69–14.94)	6.14 (3.46–11.50)	7.78 (4.60–14.39)	11.59 (7.23–18.20)	11.27 (5.51–18.57)	7.65 (4.50–14.24)
ANC × 10^3^ (cells/mm^3^)	9.44 (6.17–14.44)	8.46 (5.16–12.39)	9.46 (6.55–14.56)	10.45 (7.24–15.69)	8.02 (3.56–11.79)	10.23 (6.70–14.91)
ALC × 10^3^ (cells/mm^3^)	1.07 (0.69–1.67)	1.27 (0.83–1.88)	1.10 (0.75–1.64)	0.83 (0.54–1.31)	0.69 (0.46–0.94)	1.26 (0.84–1.85)
Hemoglobin (g/dL)	11.60 (9.90–13.50)	11.80 (10.00–13.50)	11.70 (10.03–13.60)	11.50 (9.88–13.10)	10.75 (9.40–12.30)	12.00 (10.30–13.70)
White blood cells (cells/µL)	12.60 (8.80–17.80)	11.20 (7.80–16.05)	13.05 (9.43–18.62)	13.35 (9.40–18.50)	10.35 (6.52–14.72)	13.20 (9.70–18.50)
Platelets × 10^3^ (cells/mm^3^)	223 (162–302)	222 (165–300)	219 (158–298)	230 (166–313)	190 (124–254)	237 (177–308)
1-yr mortality	276 (26.2%)	54 (15.4%)	87 (24.8%)	135 (38.6%)	121 (44.0%)	155 (19.9%)

^*^Proportion or median (IQR)

^**^Maximum value within 24 h of ICU admission

*NLR* neutrophil-to-lymphocyte ratio, *BMI* body mass index, *COPD* chronic obstructive pulmonary disease, *ICU* intensive care unit, *ANC* absolute neutrophil count, *ALC* absolute lymphocyte count

Post-hospital mortality (26.2% overall) increased across NLR tertiles (log-rank *p* < 0.005; 15–39%) and in adjusted models (Fig. 1A). Risk appeared more strongly associated with lymphopenia than neutrophilia, although comparisons were limited. Lymphopenia was associated with higher 1-year mortality (adjusted HR 1.79, 95% CI 1.40–2.29) (Fig. 1B). Compared with an ALC of 2500 cells/mm^3^, adjusted risk increased substantially below 1000 cells/mm^3^ (Fig. 1C). Among 777 non-lymphopenic patients, higher NLR remained associated with mortality, although attenuated (Fig. 1D). Associations were consistent regardless of malignancy status.

In this single-center cohort, pre-discharge NLR and lymphopenia were associated with 1-year mortality. NLR stratified risk among patients with normal ALC, suggesting prognostic value beyond laboratory-defined cut points. However, residual confounding is possible, and findings should be interpreted cautiously.

Biomarkers stratifying long-term risk after critical illness may help target post-hospital follow-up and therapy, although this remains speculative. NLR mechanisms cannot be inferred here, but prior evidence suggests risk is largely driven by the lymphocyte component [2]. NLR and lymphopenia were associated with age, comorbidities, and acute illness severity. These findings extend a prior study in the US Veterans Affairs healthcare system of sepsis survivors [5], but require validation.

Limitations include a modest sample size and a substantial proportion of NLR missingness (presumably reflecting non-ascertainment in the database and non-testing) risking selection bias—findings are exploratory; confirmatory studies are needed before clinical application. Post-hospital mortality completeness is uncertain, although observed rates were consistent with prior studies [1]. Findings are hypothesis generating and require confirmation in large prospective studies.

## Data Availability

The datasets analyzed during the current study are available from the corresponding author on reasonable request.
